# Factoring a 2 x 2 contingency table

**DOI:** 10.1371/journal.pone.0224460

**Published:** 2019-10-25

**Authors:** Stanley Luck

**Affiliations:** Science, Technology and Research Institute of Delaware, Wilmington, DE, United States of America; Universita degli Studi di Genova, ITALY

## Abstract

We show that a two-component proportional representation provides the necessary framework to account for the properties of a 2 × 2 contingency table. This corresponds to the factorization of the table as a product of proportion and diagonal row or column sum matrices. The row and column sum invariant measures for proportional variation are obtained. Geometrically, these correspond to displacements of two point vectors in the standard one-simplex, which are reduced to a center-of-mass coordinate representation, $\left(\delta ,\mu \right)\in R^{2}$. Then, effect size measures, such as the odds ratio and relative risk, correspond to different perspective functions for the mapping of (*δ*, *μ*) to $R^{1}$. Furthermore, variations in *δ* and *μ* will be associated with different cost-benefit trade-offs for a given application. Therefore, pure mathematics alone does not provide the specification of a general form for the perspective function. This implies that the question of the merits of the odds ratio versus relative risk cannot be resolved in a general way. Expressions are obtained for the marginal sum dependence and the relations between various effect size measures, including the simple matching coefficient, odds ratio, relative risk, Yule’s *Q*, *ϕ*, and Goodman and Kruskal’s *τ*_*c*|*r*_. We also show that Gini information gain (IG_*G*_) is equivalent to *ϕ*^2^ in the classification and regression tree (CART) algorithm. Then, IG_*G*_ can yield misleading results due to the dependence on marginal sums. Monte Carlo methods facilitate the detailed specification of stochastic effects in the data acquisition process and provide a practical way to estimate the confidence interval for an effect size.

## Introduction

In research with contingency tables, the ability to compare experimental results from different studies is essential for studying the dependence between categorical variables and how it is maintained. However, the data acquisition is controlled by sample size parameters that appear as row and column sums for the various categories. Association coefficients that are not adjusted for unbalanced sample size can differ between tables even if the underlying system response is unchanged [1, 2]. The dependence of the *ϕ* coefficient on the margins led to the development of the normalized form, *ϕ*/*ϕ*_*max*_ [3, 4]. Recently, VanLiere and Rosenberg investigated the allele frequency dependence of the *r*^2^ linkage disequilibrium measure [5]; note that *ϕ* and *r* refer to the same coefficient. Olivier and Bell discussed the limitations of the *ϕ* coefficient and proposed effect size thresholds for the odds ratio because it is a measure that is “not problematic” [6]. The odds ratio is invariant to scaling of rows or columns, but there is continuing debate on the merits of the odds ratio versus the relative risk [7–10]. Warrens [11] showed that members of the general family of association coefficients that are linear transformations of the simple matching coefficient do not satisfy all three desiderata for a well-behaved coefficient. The lack of consensus on the utility of the many alternative effect size measures [11, 12] led us to consider whether there might be a core set of principles and elementary properties for 2 × 2 tables that might broadly apply. In this paper, we review coordinate systems for representing proportional variation in a 2 × 2 table, which corresponds to a two-component system of point vectors in the standard one-simplex with two degrees of freedom. Then, we examine the equivalence class of tables induced by an odds ratio. The scaling invariance corresponds to a diagonal symmetry such that an odds ratio does not possess a simple interpretation in terms of proportional effects. We discuss the connections between proportion difference, odds ratio, Yule’s *Q*, and relative risk and show that an effect size statistic is more generally regarded as a perspective function, i. e., a linear fractional transformation [13] of proportional variation. A contingency table factors into a product of proportion and diagonal row or column sum matrices. Rows and columns of the proportion matrix correspond to different representations of the relation between categorical variables. Therefore, a 2 × 2 table is associated with four different forms of proportional variation. Together, these constitute the full implementation of the Goodman and Kruskal proposal that adjustment for unbalanced sample size is needed in the estimation of effect size [2]. Various forms of stochastic effects can affect a data acquisition process, so a 2 × 2 table is associated with a distribution. We discuss the use of Monte Carlo methods as a practical way to simulate a distribution of tables and estimate the confidence interval for an effect size. Finally, our interest in effect size measures developed in the course of plant breeding research at DuPont to identify agriculturally beneficial genetic variation in maize [14]. These studies involved high-dimensional search to assess linkage disequilibrium and genome-wide association (GWAS) in maize populations, including the use of the classification and regression tree (CART) algorithm. An essential step in CART is an exhaustive search over the range of each independent variable for an optimal binary partition of the response data [15, 16]. We show that the Gini information gain is equivalent to *ϕ*^2^, and we compare their behavior with a scaling invariant effect size measure using a publicly available data set. Satisfactory resolution of these longstanding issues in the application of effect size for statistics would have broad implications for high-dimensional data analysis and machine learning. The main novel contributions of this work are: 1) identification of the correspondence between factoring the 2 × 2 table and effect size, 2) identification of the four forms of proportional variation with row or column sum invariance, 3) identification of an effect size measure for a 2 × 2 table as a mapping of proportional variation for a two-component system in △^1^ × △^1^ to $R^{2}$, 4) identification of the equivalence between Gini information gain and the *ϕ* coefficient, 5) development of an improved CART association algorithm using a proportional displacement measure with correction for unbalanced sample size for the response.

## 1 Methods

### 1.1 Notation

In this work, we study the connection between odds ratio, proportion and *ϕ* for a 2 × 2 table. Our notation for the three required coordinate systems is briefly summarized here. We deviate slightly from convention and use the symbol △^1^ to designate the standard one-simplex [13] such that the dot product of a vector, **u** ∈ △^1^, with the one-vector satisfies the condition **u** ⋅ **1** = 1. Ratio vectors, (*α*, 1) and (*β*, 1), with $\alpha ,\beta \in R^{1}$ are elements of the projective line, $P^{1}$. (*α*, 1) corresponds to the proportion, *p*_*α*_ = *α*/(*α* + 1), and the proportion vector, **p**_*α*_ = (*p*_*α*_, 1 − *p*_*α*_), in △^1^. The subscript for a proportion corresponds to its $P^{1}$ coordinate. Similarly, (*β*, 1) corresponds to the proportion vector **p**_*β*_ = (*p*_*β*_, 1 − *p*_*β*_). (*a*, *b*), (*c*, *d*), (*a*, *c*), and (*b*, *d*) are vectors in $R^{2}$. (*a*, *b*) corresponds to the ratio vector, (*a*/*b*, 1), in $P^{1}$. (*a*/*b*, 1) corresponds to the proportion, $p_{a/b}=\frac{a}{b}/\left(\frac{a}{b}+1\right)$, and the proportion vector, (*p*_*a*/*b*_, *p*_*b*/*a*_) = (*p*_*a*/*b*_, 1 − *p*_*a*/*b*_), in △^1^. Ratio and proportion vectors are defined in a similar way for the other $R^{2}$ vectors. The slightly cumbersome subscript notation is necessary because we are working with proportions for both row space such as ‘*p*_*a*/*b*_’, and column space such as ‘*p*_*a*/*c*_’. However, in subscripts for marginal sum proportions the division by *N* is dropped; e. g., *p*_*a*+*c*_ = (*a* + *c*)/*N* where *N* = *a* + *b* + *c* + *d*. Ratio and proportion vectors are examples of perspective functions of the general form $P\left(u,t\right)=\frac{u}{t}$ for $u\in R^{N}$, $t\in R^{1}$, and *t* > 0 [13]. Another familiar example is normalization by the Euclidean norm, $P\left(u,||u||\right)=\frac{1}{\left||u|\right|}u$.

### 1.2 Coordinate systems for proportion and odds ratio

In this section, we discuss coordinate systems for representing binary proportional variation in categorical data analysis. For the point vector $\left(a,b\right)\in R^{2}$, the ratio corresponds to a linear fractional transformation

$$\begin{array}{cc} \frac{a}{b} & =\frac{\left(a+b)+(a-b\right)}{\left(a+b)-(a-b\right)},\\ & =\frac{1+\delta_{s}}{1-\delta_{s}}, \end{array}$$ (1)

where *δ*_*s*_ is the difference in proportion

$$\begin{array}{cc} \delta_{s} & =\frac{a-b}{a+b},\\ & =\frac{\frac{a}{b}-1}{\frac{a}{b}+1}. \end{array}$$

The ‘*s*’ designation arises from the connection with the proportional displacement, ***δ***_*s*_, between the pair of vectors (*a*, *b*) and (*b*, *a*),

$$\begin{array}{cc} \delta_{s} & =\frac{1}{a+b}[(a,b)-(b,a)],\\ & =\delta_{s}\left(1,-1\right), \end{array}$$ (2)

and the correspondence of these vectors to a diagonally ‘symmetric’ 2 × 2 table as described in Section 1.4. We will encounter several expressions of the form Eq (1), indicating that elements of projective geometry [13, 17] provide the framework for the analysis of proportional variation. Consequently, our objective is to identify vector algebraic structures for representing proportional variation in asymmetric 2 × 2 tables. They provide the framework for analyzing the relationships between binary proportion, odds ratio, Yule’s *Q*, relative risk, and *ϕ*.

**Fig 1:**
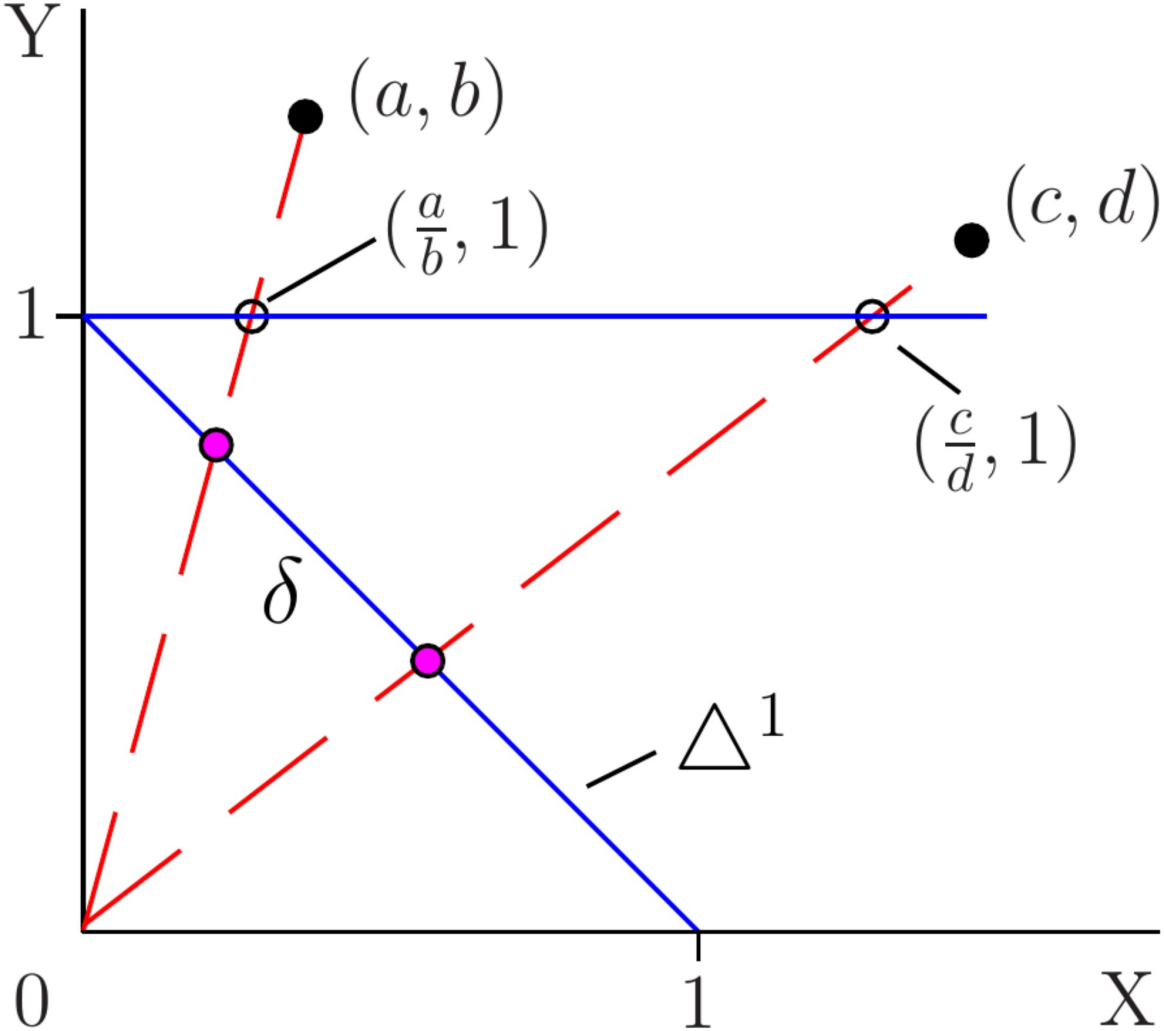
Coordinates for a two-component binary proportional system. Proportional variation for vectors, (*a*, *b*) and (*c*, *d*), is represented either as points, $\left(\frac{a}{b},1\right)$ and $\left(\frac{c}{d},1\right)$, in $P^{1}$, or as points in the standard one-simplex, △^1^. *δ* is the proportional displacement between the vectors. Proportion and ratio are related by a linear fractional transformation, as indicated by the dashed lines.

Proportional normalization of a ratio vector produces a proportion vector

$$\begin{array}{cc} \frac{1}{\frac{a}{b}+1}(\frac{a}{b},1) & =(\frac{a}{a+b},\frac{b}{a+b}),\\ & =P((a,b),a+b), \end{array}$$

which is an element of △^1^ (Fig 1). Then, a proportion vector has the form **v** = (*v*_1_, 1 − *v*_1_), with derivative *d***v** = *dv*_1_(1, −1) such that *d***v** ⋅ **1** = 0 for 0 ≤ *v*_1_ ≤ 1. In contrast, the corresponding ratio vector has the form

$$v^{\prime }=(\frac{v_{1}}{1-v_{1}},1),$$

with derivative

$$\begin{array}{c} dv^{\prime }=\frac{dv_{1}}{{\left(1-v_{1}\right)}^{2}}(1,0). \end{array}$$

Then, the difference between proportion vectors **u** and **v**

$$\begin{array}{cc} \delta & =u-v,\\ & =\left(u_{1}-v_{1}\right)\left(1,-1\right), \end{array}$$

is parameterized by a single parameter, *u*_1_ − *v*_1_, and variation in binary proportion corresponds to translation in △^1^. The difference between ratio vectors is also parameterized by a single parameter,

$$\begin{array}{cc} \delta^{\prime } & =u^{\prime }-v^{\prime },\\ & =\frac{\left(u_{1}-v_{1}\right)}{\left(1-u_{1}\right)\left(1-v_{1}\right)}\left(1,0\right). \end{array}$$

Therefore, △^1^ and $P^{1}$ correspond to different constraints in representing proportional variation. However, the order of categories in a contingency table is arbitrary, and it is not possible to identify a unique category that should serve as the perspective coordinate for a ratio. This introduces ambiguity, as we will see later in the discussion of the odds ratio. On the other hand, in factoring out the effects of marginal sums, the △^1^ representation provides an important function in the analysis of 2 × 2 tables.

Now, we discuss the representation of a two-component system of binary proportions in △^1^ and $P^{1}$ coordinate systems, and describe intrinsic properties of various effect size measures. The formulae take on a more compact, intuitive form because scaling invariance is built-in. The algebraic intuition gained here helps in comprehending the more cumbersome expressions obtained later using the $R^{2}$ representation. The exception is the *ϕ* coefficient, which does not possess a △^1^ representation due to the lack of scaling invariance (section 1.4). In particular, we discuss properties of the odds ratio, *ω* = *β*/*α*, where *α*, *β* ≥ 0, corresponding to (*α*, 1) and (*β*, 1) on the $P^{1}$ line, respectively. Then, relative risk is defined as *ρ* = *p*_*β*_/*p*_*α*_, where *p*_*β*_ = *β*/(*β* + 1) and *p*_*α*_ = *α*/(*α* + 1). The corresponding proportional basis consists of **p**_*α*_ = (*p*_*α*_, 1 − *p*_*α*_) and **p**_*β*_ = (*p*_*β*_, 1 − *p*_*β*_). Next, we introduce the center-of-mass basis

$$\begin{array}{cc} \delta_{\beta -\alpha } & =\frac{1}{2}\left(p_{\beta }-p_{\alpha }\right),\\ & =\delta (1,-1),\\ \mu_{\alpha +\beta } & =\frac{1}{2}\left(p_{\alpha }+p_{\beta }\right),\\ & =(\mu ,1-\mu ), \end{array}$$

with the parameters $\delta =\frac{p_{\beta }-p_{\alpha }}{2}$ and $\mu =\frac{p_{\alpha }+p_{\beta }}{2}$; note that the alternative basis ***δ***_*α*−*β*_ and ***μ***_*α*+*β*_ would also suffice. Then, variation is represented by the two-parameter vector (*δ*, *μ*), reflecting the fact that there are two degrees of freedom. Using the relations $\alpha =\frac{p_{\alpha }}{1-p_{\alpha }}$, $\beta =\frac{p_{\beta }}{1-p_{\beta }}$, *p*_*α*_ = *μ* − *δ* and *p*_*β*_ = *μ* + *δ*, we obtain

$$\begin{array}{cc} \omega & =\frac{p_{\beta }-p_{\alpha }p_{\beta }}{p_{\alpha }-p_{\alpha }p_{\beta }},\\ & =\frac{\delta^{2}+\mu \left(1-\mu \right)+\delta }{\delta^{2}+\mu \left(1-\mu \right)-\delta }. \end{array}$$ (3)

Then, we introduce Yule’s *Q* [1] to obtain

$$\begin{array}{cc} Q & =\frac{\omega -1}{\omega +1},\\ & =\frac{\delta }{\delta^{2}+\mu \left(1-\mu \right)}. \end{array}$$ (4)

Similarly, the relative risk is expressed as

$$\begin{array}{cc} \rho & =\frac{p_{\beta }}{p_{\alpha }},\\ & =\frac{\mu +\delta }{\mu -\delta }, \end{array}$$ (5)

and the ratio difference is expressed as

$$\begin{array}{cc} \beta -\alpha & =\frac{p_{\beta }-p_{\alpha }}{\left(1-p_{\beta }\right)\left(1-p_{\alpha }\right)},\\ & =\frac{\delta }{1+\mu \left(\mu -2\right)-\delta^{2}}. \end{array}$$ (6)

Inspection of Eqs (3–6) shows that the odds ratio and relative risk correspond to linear fractional transformations of proportional variation, and an effect size statistic corresponds to a perspective function *P*((*δ*, *μ*), *t*) = (*δ*/*t*, *μ*/*t*), where *t* is a polynomial function of *δ* and *μ*. However, algebraic considerations alone are not sufficient to explain why a particular form might be preferred for *t* or to provide operational interpretations for the different perspective normalizations in Eqs (4–6). In his 1912 paper, Yule remarked that the *Q* coefficient has the merit of possessing a simple form “but the demerit of not possessing an equal simplicity of interpretation” [1]. Given the lack of an interpretation for the different normalizations, we find that Yule’s remark also extends to the odds ratio and relative risk. Furthermore, rearranging Eqs (4) and (5) gives the corresponding relations

$$\delta^{2}-\frac{\delta }{Q}+\mu \left(1-\mu \right)=0,$$ (7)

**Fig 2:**
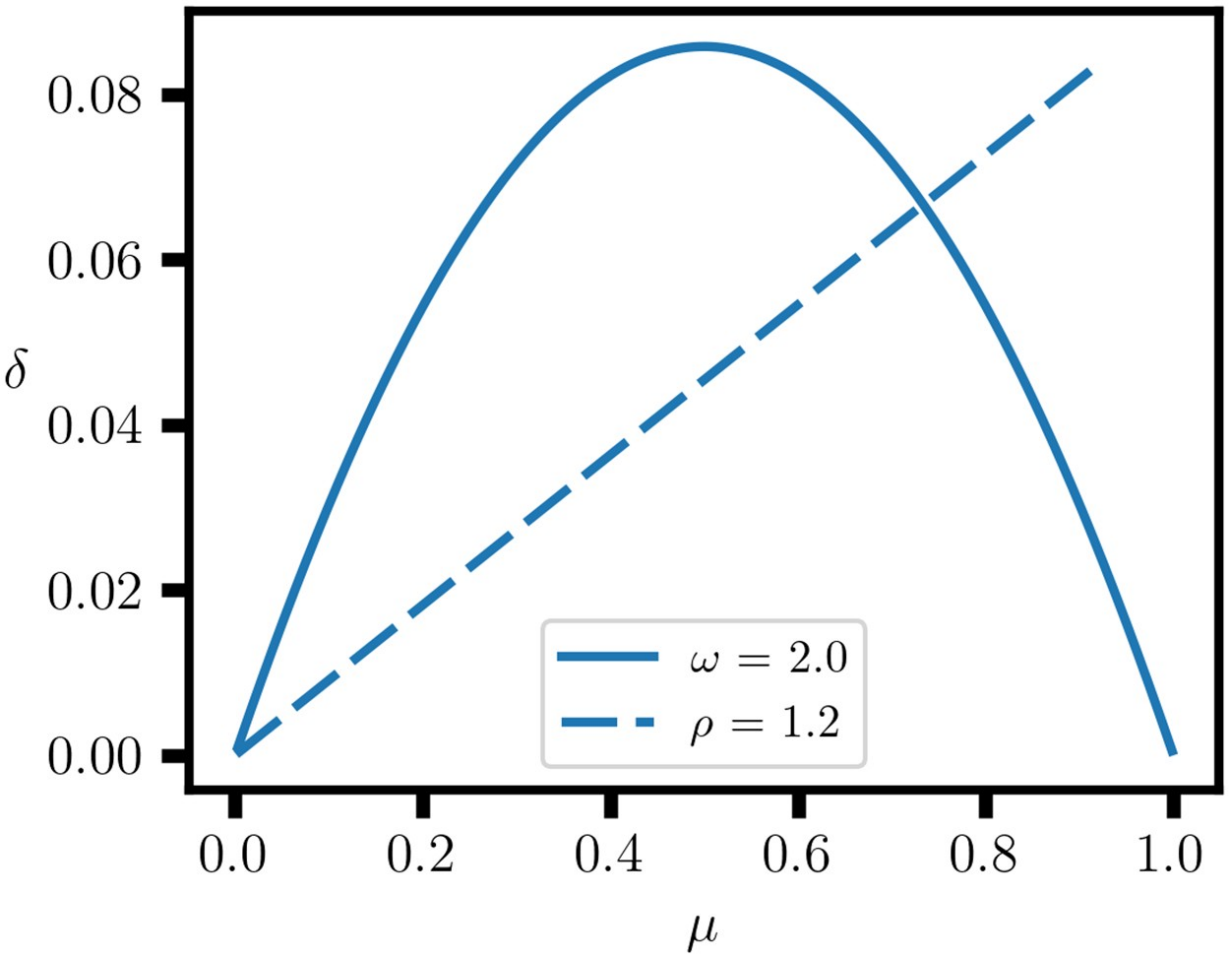
Center-of-mass coordinates for a two-component biproportional system. In the △^1^ representation, the center-of-mass coordinates are *μ* = (*p*_*α*_ + *p*_*β*_)/2 and *δ* = (*p*_*β*_ − *p*_*α*_)/2. The proportional variation, (*μ*, *δ*), for fixed odds ratio, *ω* = 2, and relative risk, *ρ* = 1.2, are shown. The odds ratio and relative risk are perspective functions of the center-of-mass coordinates.

δ(ρ+1)−μ(ρ−1)=0, (8)

with 0 ≤ *μ* − *δ* ≤ 1 and 0 ≤ *μ* + *δ* ≤ 1. Each of the four forms of proportional variation identified in the section 1.3 satisfies these relations. Thus, there are a range of values of (*δ*, *μ*) for a fixed value of either *Q*, or *ρ* (Fig 2). This ambiguity in proportional effects explains why the question of the merits of the odds ratio versus relative risk is still not resolved [18, 19]. A more precise approach would take into account the two-dimensional nature of the proportional variation, which could involve separate thresholds for *δ* and *μ*. In any case, the specification of a perspective function should be based on the assessment of cost-benefit trade-offs for variations in *δ* and *μ*, which will depend on the particular application.

### 1.3 Decomposition of proportional variation for a 2 × 2 contingency table

In this section, the two-component framework is used in the analysis of proportional variation for a 2 × 2 table (Table 1). We are particularly concerned with the confounding effect of the row and column sums in the formulation of association measures [2, 5, 11]. Each marginal sum corresponds to a categorical sample size that is determined by experimental procedure. Suppose the first row of Table 1 is multiplied by a number *k* to reflect a change in sample size; then, (*a*, *b*) ↦ (*ka*, *kb*). Then, the simple matching coefficient [11], *s*_*M*_, is expressed as

$$s_{M}=\frac{ka+d}{k(a+b)+c+d},$$

which is not invariant to scaling by *k*. Alternatively, each marginal sum serves as a proportional normalization factor; e. g., *P*((*a*, *b*), *a* + *b*). Then, *s*_*M*_ can be expressed as the weighted sum of proportions

$$\begin{array}{cc} s_{M} & =\frac{a+c}{a+b+c+d}\frac{a}{a+c}+\frac{b+d}{a+b+c+d}\frac{d}{b+d}\\ & ≕x_{a+c}p_{a/c}+x_{b+d}p_{d/b} \end{array}$$ (9)

$$\begin{array}{cc} & =\frac{a+b}{a+b+c+d}\frac{a}{a+b}+\frac{c+d}{a+b+c+d}\frac{d}{c+d}\\ & ≕x_{a+b}p_{a/b}+x_{c+d}p_{d/c} \end{array}$$ (10)

for columns or rows, respectively. The proportions are invariant to scaling of either rows or columns, but the corresponding weights (*x*_*i*_) are not because the overall sum, *a* + *b* + *c* + *d*, does not distinguish between row or column sums. Therefore, *s*_*M*_ can differ between two tables because of differences in sample size even though the underlying system response properties might be unchanged. Warrens [11] has shown that members of the general family of coefficients that are linear transformations of *s*_*M*_ do not satisfy the criteria for a well-behaved coefficient. As discussed by Goodman and Kruskal [2], dependence on sample size parameters complicates the interpretation of association coefficients. The concepts discussed in this paper support their proposal that normalization to adjust for unbalanced sample sizes is necessary.

**Table 1:** The 2 × 2 contingency table.

	Column 1	Column 2	Row sum
**Row** 1	a	b	*a* + *b*
**Row** 2	c	d	*c* + *d*
**Column sum**	*a* + *c*	*b* + *d*	

Observed counts, (*a*, *b*, *c*, *d*), of the joint occurrence of categorical events are listed in the table. The sample size parameters for the classifications appear as the row sums, (*a* + *b*, *c* + *d*), and column sums, (*a* + *c*, *b* + *d*). The column and row sums are linearly related, and each sum serves as a scale factor for proportion.

The invariance of the odds ratio to scaling of either rows or columns is expressed as

$$\begin{array}{c} \omega \frac{bc}{k}-\frac{ad}{k}=0, \end{array}$$ (11)

**Fig 3:**
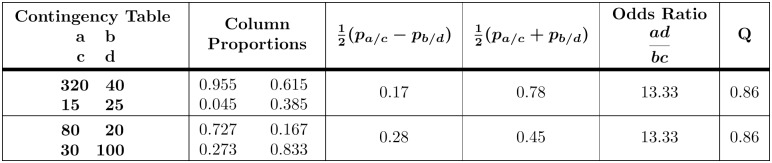
Contingency tables with fixed odds ratio. While the odds ratio, $\omega =\frac{ad}{bc}$, is fixed in these tables, the proportions are not. The Yule *Q* statistic is also invariant because it is related to *ω* by the linear fractional transformation $Q=\frac{\omega -1}{\omega +1}$.

*k* > 0. This expression remains valid if either *bc* ↦ *cb* or *ad* ↦ *da*. Thus, the odds ratio does not distinguish between ratios for rows and columns, $\omega =\frac{a}{b}\frac{d}{c}=\frac{a}{c}\frac{d}{b}$ [18, 20], which introduces ambiguity with respect to proportional effects. Consider the equivalence class of tables obtained by unitary scaling of the diagonal elements (‘u-scaling’),

$$\begin{array}{c} \omega \frac{b}{j}\frac{c}{j^{-1}}-\frac{a}{k^{-1}}\frac{d}{k}=0, \end{array}$$

with *j*, *k* > 0 (Table 2). The two numerical examples of such tables shown in Fig 3 demonstrate that while the odds ratio and *Q* are invariant, the proportions are not. Furthermore, in the special case where $j=\sqrt{b/c}$ and $k=\sqrt{d/a}$, the row and column sums are equalized due to the geometric averaging of the diagonal elements, and the Yule symmetric table (Table 3) is obtained. This table serves as the basis for Yule’s *ω* coefficient [1], also known as the coefficient of colligation [21]. However, row and column sums are linearly related by a column proportion matrix

$$\begin{array}{cc} \left(\begin{array}{c} a+b\\ c+d \end{array}\right) & =(\begin{array}{cc} \frac{a}{a+c} & \frac{b}{b+d}\\ \frac{c}{a+c} & \frac{d}{b+d} \end{array})(\begin{array}{c} a+c\\ b+d \end{array}). \end{array}$$

This linear relation is not preserved by u-scaling because of the mixing of effects between rows and columns (Table 2), so the odds ratio by itself is not suitable as an effect size measure. The linear relation also implies that row and column sums play equal roles as sample size parameters directly or indirectly, and that either rows or columns can be equalized, but not both simultaneously. It is necessary to choose between rows or columns in conditioning a contingency table for unbalanced sample sizes.

**Table 2:** Diagonal scaling invariance of the odds ratio.

	Column 1	Column 2	Row sum
**Row 1**	*ka*	$\frac{b}{j}$	$ka+\frac{b}{j}$
**Row 2**	*jc*	$\frac{d}{k}$	$jc+\frac{d}{j}$
**Column sum**	*ka* + *jc*	$\frac{b}{j}+\frac{d}{k}$	

The odds ratio does not distinguish between rows and columns, $\omega =\frac{a}{b}\frac{d}{c}=\frac{a}{c}\frac{d}{b}$, so the odds ratio is invariant to unitary scaling of the diagonal elements with *j* > 0, *k* > 0.

**Table 3:** The Yule symmetric table.

	Column 1	Column 2	Row sum
**Row 1**	$\sqrt{ad}$	$\sqrt{bc}$	$\sqrt{ad}+\sqrt{bc}$
**Row 2**	$\sqrt{bc}$	$\sqrt{ad}$	$\sqrt{ad}+\sqrt{bc}$
**Column sum**	$\sqrt{ad}+\sqrt{bc}$	$\sqrt{ad}+\sqrt{bc}$	

The equivalence class for an odds ratio includes this symmetric table obtained by geometric averaging of the diagonal elements; $j=\sqrt{b/c}$ and $k=\sqrt{d/a}$ in Table 2. This results in the equalization of column and row sums and a loss of information.

A self-consistent representation of proportional variation must account for the scaling invariance of the odds ratio. Therefore, our objective is to obtain a decomposition of the odds ratio in terms of elementary proportions by conditioning for the effect of the marginal sums. Consider scaling of the expression *ωbc* − *ad* = 0 by column sums to obtain the fractional representation

$$\begin{array}{cc} \omega \frac{b}{n_{1}\left(b+d\right)}\frac{c}{n_{2}\left(a+c\right)}-\frac{a}{n_{1}\left(a+c\right)}\frac{d}{n_{2}\left(b+d\right)} & =0, \end{array}$$ (12)

where *n*_1_ and *n*_2_ are normalization factors for the subsequent conversion to proportion vectors. Since there are two ways to express the odds ratio as a product of ratios, there are also two ways to group the fractional products to form proportion vectors. The standard grouping is formed from the columns of the table with *n*_1_ = *n*_2_ = 1 to obtain the two vectors

$$\begin{array}{c} (\frac{a}{a+c},\frac{c}{a+c}),(\frac{b}{b+d},\frac{d}{b+d}). \end{array}$$ (13)

However, we also obtain a second pair of vectors formed from the rows with $n_{1}=\frac{a}{a+c}+\frac{b}{b+d}$ and $n_{2}=\frac{c}{a+c}+\frac{d}{b+d}$ yielding

$$\begin{array}{c} (\frac{a}{a+b\frac{a+c}{b+d}},\frac{b}{b+a\frac{b+d}{a+c}}),(\frac{c}{c+d\frac{a+c}{b+d}},\frac{d}{d+c\frac{b+d}{a+c}}). \end{array}$$ (14)

The proportions in both Eqs (13) and (14) are invariant to scaling of columns, as required. The second form of proportional variation corresponds to an effect size measure with the normalization needed for experimental work, and has not been previously mentioned in the effect size literature to the best of my knowledge. Proportion vectors invariant to the scaling of rows are obtained in a similar way. A more concise way to obtain the proportion vectors is to observe that a matrix can be factored as a product of a diagonal column sum (**M**_csum_) or a row sum (**M**_rsum_) and proportion matrices, **P**_csum,c|r_ or **P**_rsum,c|r_, respectively.

$$\begin{array}{cc} \left(\begin{array}{cc} a & b\\ c & d \end{array}\right) & =(\begin{array}{cc} n_{1} & 0\\ 0 & n_{2} \end{array})(\begin{array}{cc} \frac{a}{n_{1}\left(a+c\right)} & \frac{b}{n_{1}\left(b+d\right)}\\ \frac{c}{n_{2}\left(a+c\right)} & \frac{d}{n_{2}\left(b+d\right)} \end{array})(\begin{array}{cc} a+c & 0\\ 0 & b+d \end{array}),\\ & =N_{\text{csum},c|r}P_{\text{csum},c|r}M_{\text{csum}}, \end{array}$$ (15)

$$\begin{array}{cc} & =(\begin{array}{cc} a+b & 0\\ 0 & c+d \end{array})(\begin{array}{cc} \frac{a}{n_{1}\left(a+b\right)} & \frac{b}{n_{2}\left(a+b\right)}\\ \frac{c}{n_{1}\left(c+d\right)} & \frac{d}{n_{2}\left(c+d\right)} \end{array})(\begin{array}{cc} n_{1} & 0\\ 0 & n_{2} \end{array}),\\ & =M_{\text{rsum}}P_{\text{rsum},c|r}N_{\text{rsum},c|r}. \end{array}$$ (16)

**Fig 4:**
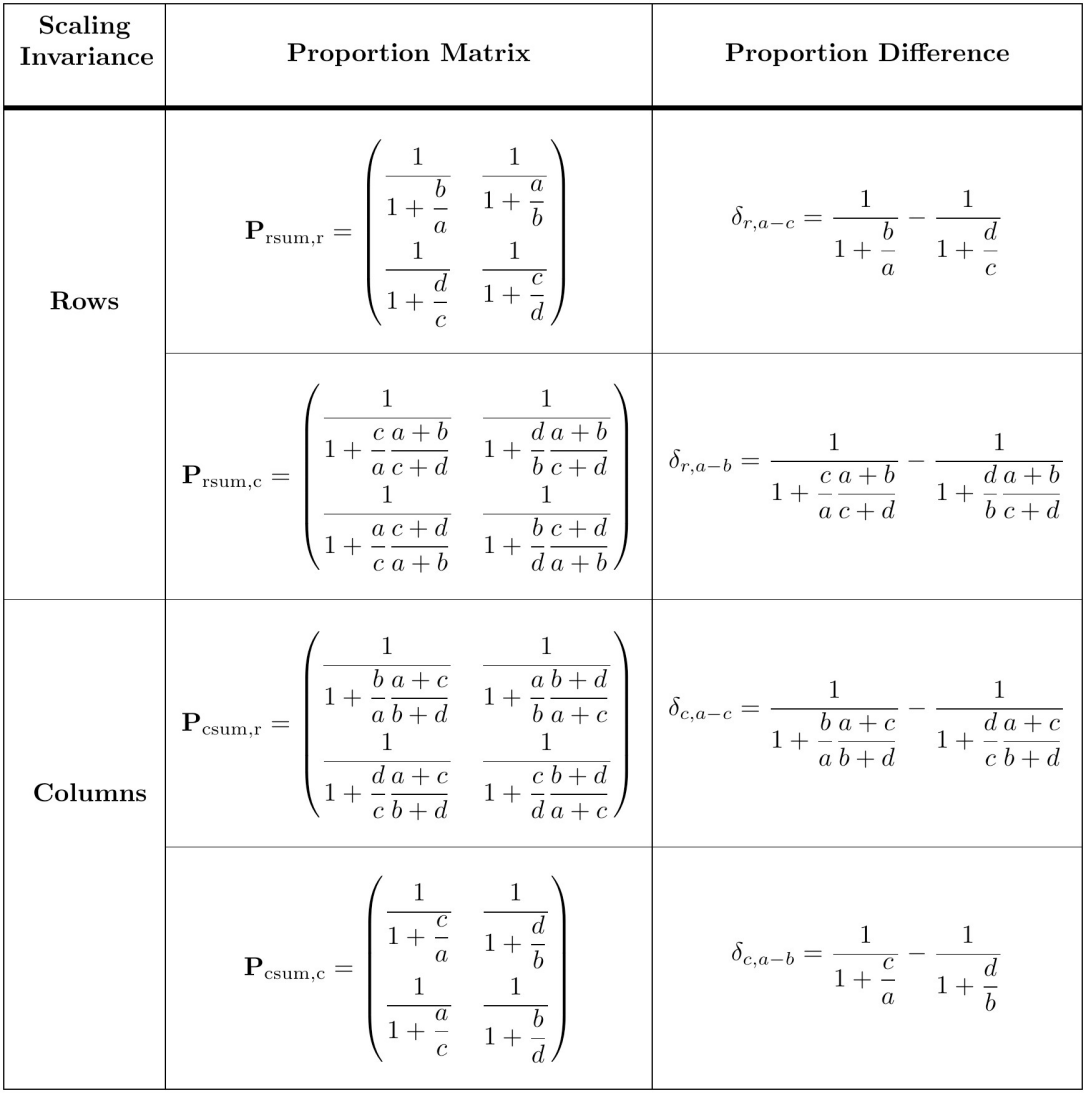
Four forms of proportional variation for a 2 × 2 table. Separate proportion matrices are obtained in factoring a 2 × 2 matrix for scaling by the column sum (csum) or the row sum (rsum). Columns and rows of a proportion matrix correspond to different representations of the relationship between categorical variables.

The **N**_csum,c|r_ and **N**_rsum,c|r_ proportion normalization factors provide the different scaling structures (Eq 12) needed for column and row proportion matrices, which correspond to different projective representations of the relationship between variables (Fig 4). The standard protocol is to equalize the marginal sums for the response or dependent variable, and calculate the response effect size for variation of the treatment or independent variable. Depending on whether the response variable is listed in columns or rows, the corresponding representation would be either **P**_csum,r_ or **P**_rsum,c_, respectively. Examples of corresponding proportion difference measures, *δ*_*c*,*a*−*c*_ and *δ*_*r*,*a*−*b*_, are also shown in Fig 4. Our subscript notation is explained by the following example,

$$\begin{array}{cc} \delta_{r,a-b} & =\frac{1}{1+\frac{c}{a}\frac{a+b}{c+d}}-\frac{1}{1+\frac{d}{b}\frac{a+b}{c+d}},\\ & =\frac{a}{a+c\frac{a+b}{c+d}}-\frac{b}{b+d\frac{a+b}{c+d}}. \end{array}$$

Thus, *δ*_*r*,*a*−*b*_ corresponds to the difference between ‘*a*’ and ‘*b*’ elements of the **P**_rsum,c_ proportion matrix. Then, calculation of an effect size requires the specification of a perspective function for mapping the relevant (*δ*, *μ*) vector to $R^{1}$ (Section 1.2). Proper practice also requires that an effect size estimate must be qualified by a confidence interval (Section 1.5).

### 1.4 The *ϕ* coefficient

In this section, we discuss why *ϕ* does not serve as a well-behaved effect size measure and further explain the connection between *δ*_*s*_ and diagonally symmetric 2 × 2 tables. The *ϕ* coefficient is of particular importance in GWAS because it serves as a standard measure of linkage disequilibrium between molecular markers [3, 5]. The popularity of *ϕ* is due to its correspondence with Pearson’s correlation coefficient. Binary {0, 1} representations are invoked for the categorical variables, then the correlation coefficient formula is applied to obtain

$$\phi =\frac{ad-bc}{\sqrt{\left(a+b)(c+d)(a+c)(b+d\right)}},$$ (17)

which is also often referred to as ‘*r*’. However, the limitations of *ϕ* as an association measure are well known [3, 5, 6, 11, 22]. Alternatively, *ϕ* is obtained from the relation with Pearson’s chi-squared statistic, *χ*^2^ = (*a* + *b* + *c* + *d*)*ϕ*^2^ [3, 23], which also averages over rows and columns resulting in confounding effects. Introducing the ratio product for marginal sums,

$$\omega_{M}=\frac{\left(a+b)(c+d\right)}{\left(a+c)(b+d\right)},$$ (18)

*ϕ* can be written as the row sum factorization

$$\begin{array}{cc} \phi & =\sqrt{\omega_{M}}\frac{ad-bc}{\left(a+b)(c+d\right)},\\ & =\sqrt{\omega_{M}}(\frac{a}{a+b}-\frac{c}{c+d}),\\ & =\frac{1}{\sqrt{\left(a+c)(b+d\right)}}(\sqrt{\frac{c+d}{a+b}}a-\sqrt{\frac{a+b}{c+d}}c), \end{array}$$

which corresponds to the scaling of **P**_rsum,r_

$$\sqrt{\omega_{M}}(\begin{array}{cc} \frac{a}{a+b} & \frac{b}{a+b}\\ \frac{c}{c+d} & \frac{d}{c+d} \end{array})=\frac{1}{\sqrt{\left(a+c)(b+d\right)}}(\begin{array}{cc} \sqrt{\frac{c+d}{a+b}}a & \sqrt{\frac{c+d}{a+b}}b\\ \sqrt{\frac{a+b}{c+d}}c & \sqrt{\frac{a+b}{c+d}}d \end{array}).$$

Therefore, *ϕ* corresponds to u-scaling of the 2 × 2 table with $j=\sqrt{\frac{a+b}{c+d}}$ and $k=\sqrt{\frac{c+d}{a+b}}$. Alternatively, the column sum factorization for *ϕ* is

$$\begin{array}{cc} \phi & =\frac{1}{\sqrt{\omega_{M}}}(\frac{a}{a+c}-\frac{b}{b+d}),\\ & =\frac{1}{\sqrt{\left(a+b)(c+d\right)}}(\sqrt{\frac{b+d}{a+c}}a-\sqrt{\frac{a+c}{b+d}}b), \end{array}$$

which corresponds to the u-scaling of the 2 × 2 table with $j=k=\sqrt{\frac{b+d}{a+c}}$. The following factorizations also hold:

$$\phi =\delta_{r,a-b}\frac{\left(a+c\frac{a+b}{c+d})(b\frac{c+d}{a+b}+d\right)}{\sqrt{\left(a+b)(c+d)(a+c)(b+d\right)}},$$ (19)

$$\begin{array}{cc} & ≕\;\delta_{r,a-b}M_{r,a-b}, \end{array}$$ (20)

$$\begin{array}{cc} & =\delta_{c,a-c}\frac{\left(a+b\frac{a+c}{b+d})(c\frac{b+d}{a+c}+d\right)}{\sqrt{\left(a+b)(c+d)(a+c)(b+d\right)}}, \end{array}$$ (21)

$$\begin{array}{cc} & ≕\;\delta_{c,a-c}M_{c,a-c}. \end{array}$$ (22)

Consequently, each proportion difference, *δ*_*i*_, is associated with a factorization *ϕ* = *M*_*i*_*δ*_*i*_, where *M*_*i*_ depends on marginal sums. Therefore, *ϕ* corresponds to a weighted average of the *δ*_*i*_. The multiplication of row and column sums together in each *M*_*i*_ has a compounding effect because the sums are not independent.

Consider a diagonally symmetric 2 × 2 table with *d* = *a* and *c* = *b* in Table 1, and equal row and column sums. Then, Eq (12) becomes

$$\frac{\omega b^{2}-a^{2}}{{\left(a+b\right)}^{2}}=0,$$

which corresponds to the proportion vectors $\frac{1}{a+b}\left(a,b\right)$ and $\frac{1}{a+b}\left(b,a\right)$, and proportion difference *δ*_*s*_ (Eq 2). Since $\frac{1}{a+b}\left[\left(a,b\right)+\left(b,a\right)\right]=\left(1,1\right)$, the △^1^ coordinates are $\left(\delta_{s},\frac{1}{2}\right)$, so there is only one degree of freedom. Thus, there is a correspondence between 2 × 1 tables [23] and diagonally symmetric 2 × 2 tables. However, *M*_*i*_ = 1 for diagonally symmetric tables, and Eq (17) simplifies to give *ϕ*_*s*_ = *δ*_*s*_. Thus, *δ*_*s*_ and *ϕ*_*s*_ are equivalent measures of proportional variation. Conversely, the *δ*_*i*_ in Fig 4 can be regarded as constituting an extension of *ϕ*_*s*_ to asymmetric tables. The *ϕ* coefficient per se does not account for the loss of symmetry when *M*_*i*_ ≪ 1, because it does not distinguish between the *δ*_*i*_. However, when *M*_*i*_ ≊ 1 the four expressions collapse into one or nearly so, and the values of *ϕ* and *δ*_*i*_ will be approximately the same. This includes the case where either *b* = *c* = 0 or *a* = *d* = 0 resulting in a diagonal 2 × 2 table. The connection with *ϕ* suggests that Cohen’s recommendations of effect sizes of 0.1, 0.3 and 0.5 for small, medium, and large effects, respectively, for *ϕ* [6, 24] can also be invoked for the various forms of *δ*_*i*_, but this assumes that the *μ*_*i*_ coordinate is irrelevant.

### 1.5 Confidence interval for proportional effects

**Fig 5:**
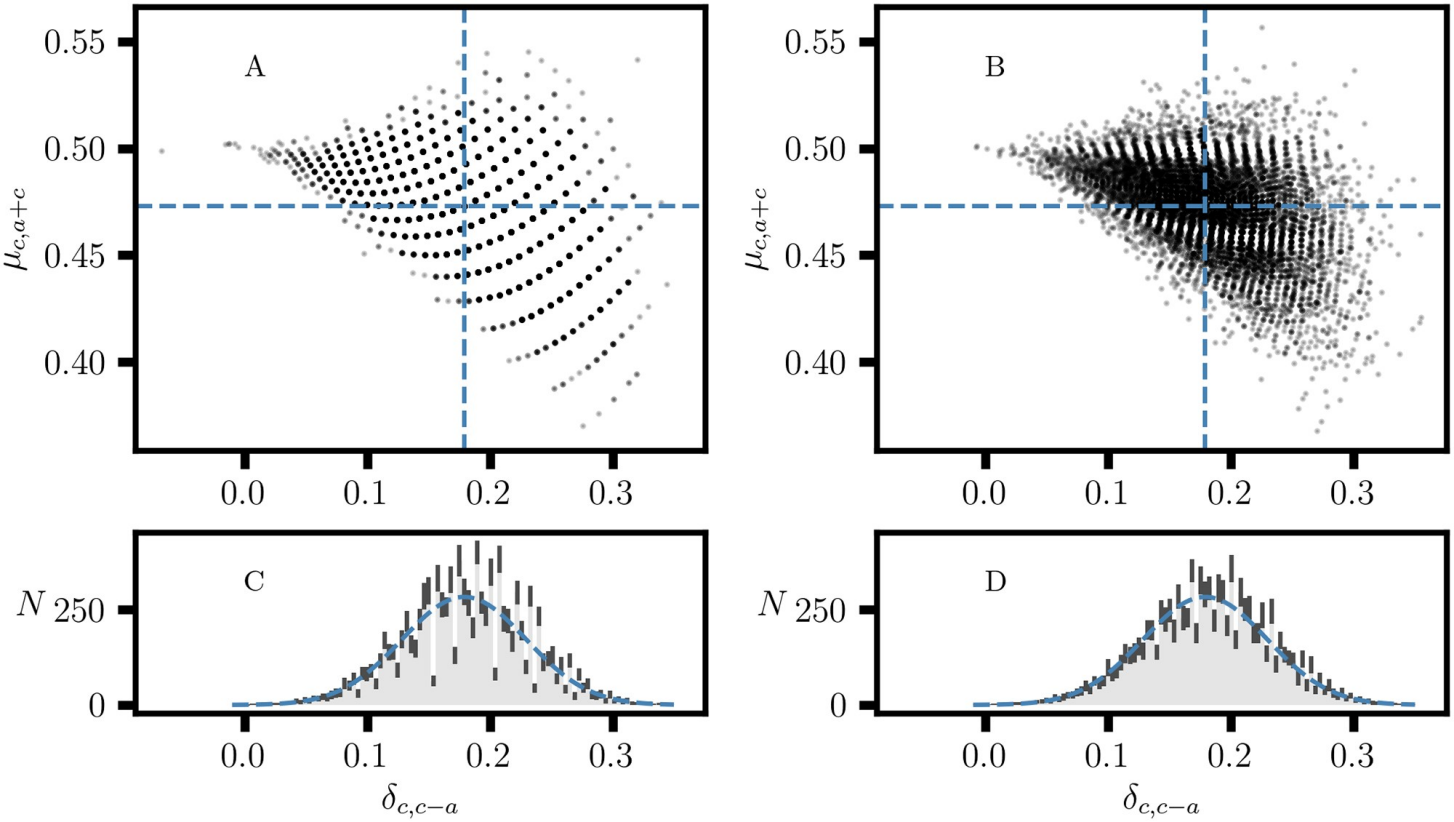
Two sets of constrained Monte Carlo (MC) simulations of the distribution of proportional variation, (*δ*_*c*,*c*−*a*_, *μ*_*c*,*c*+*a*_), for a 2 × 2 table with *a*, *b*, *c*, *d* = [10, 30, 30, 20]. A,C: MC with fixed column sums, *n*_1_ = *a* + *c* and *n*_2_ = *b* + *d*. B,D: MC with fixed overall sum *N* = *a* + *b* + *c* + *d*. A,B: Data for 10000 MC tables. The dashed lines indicate the expected values, *δ*_*c*,*c*−*a*_ = 0.18 and *μ*_*c*,*a*+*c*_ = 0.473. C,D: Each histogram is the mean of 64 MC runs with 10000 MC tables per run. Each whisker is the ±2 standard deviation interval. The normal distribution, *μ* = 0.18 and *σ* = 0.0506, is shown as a dashed curve.

Each step of a data acquisition process is subject to stochastic effects, and data quality can vary between data sets. Therefore, the specification of a confidence interval (CI) for the effect size is an integral part of data analysis [25, 26]. A contingency table for experimental data is associated with a distribution of tables, P(θ), and corresponding distributions for the effect size. The specification of P(θ) must be based on a realistic assessment of all sources of error and uncertainty to form an error model for the data, E(θ). For binary variables, a common approach is to estimate variance from a binomial distribution; the normal distribution is a useful approximation for large sample sizes. Then, estimating the CI for an effect size requires a propagation of error calculation, which is often not straightforward. Analytical approaches for estimating confidence intervals for ratios [27, 28], proportion and difference of two proportions [29, 30], correlation coefficients [31, 32], and odds ratios [9] are already quite involved. Fractional transformation, the bounded range, and the discrete properties of an effect size for proportional variation introduce complications that make it difficult to obtain convenient expressions for error propagation. Alternatively, Monte Carlo (MC) methods [33, 34] provide a more practical approach to estimate confidence intervals for quantities such as *δ*_*r*,*b*−*a*_ and *δ*_*c*,*c*−*a*_. In an MC simulation, a 2 × 2 MC table is obtained by generating the *N* = *a* + *b* + *c* + *d* events by making random draws according to specified sample proportions [9] and E(θ). A set of MC tables is obtained by repeating the sampling process many times; MC distributions are formed for proportions and effect size from the MC tables. Many MC runs are performed, collecting the relevant statistics for each MC distribution, including the mean, median, variance, and histogram. Finally, the degree of convergence for the MC simulation is estimated from the statistics for the MC runs. Fig 5A and 5C shows constrained MC simulations with fixed column sums *n*_1_ = *a* + *c* and *n*_2_ = *b* + *d* and sampling proportions $\frac{1}{a+c}\left(a,c\right)$ and $\frac{1}{b+d}\left(b,d\right)$, respectively. Fig 5B and 5D shows greater internal scatter because only the overall sum, *N*, is fixed, with corresponding sampling proportions $\frac{1}{N}\left(a,b,c,d\right)$. Even though the underlying distributions are discrete, the ±2*σ* interval for a normal distribution serves as a good approximation for the *δ*_*c*,*c*−*a*_ confidence interval in this example. More generally, the distribution of effect size is asymmetric which would be represented by separate confidence intervals for positive and negative deviation from the median. The advantage of the MC method is that the simulation can accommodate a detailed specification of E(θ), including heteroscedasticity [25, 35] and correction for attenuation from misclassification [35, 36]. This capability is essential in accounting for the effects of instrumental and other operational factors on the quality of data produced by a data acquisition system.

### 1.6 Decomposition of proportional effects for an *r* × *c* table

A table with more than two rows or columns is commonly referred to as an *r* × *c* table. The matrix factorization (Eqs 15 & 16) extends in a straightforward way to produce the *r* × *c* proportion matrices. For independent and dependent variables with *r* and *c* categories, respectively, proportional variation is represented as *r* points in the standard △^*c*−1^ simplex, with *r*(*c* − 1) degrees of freedom. Various multicategorical association measures have been proposed for *r* × *c* tables. However, we choose Cramer’s *V*^2^ [37, 38] as an example to illustrate the difficulties. *V*^2^ is defined as a normalization of Pearson’s *χ*^2^ such that *χ*^2^ = *n*(*q* − 1)*V*^2^, where *n* is the total event count and *q* = *min*(*r*, *c*). *V* is equivalent to *ϕ* for 2 × 2 tables. Similarly, it is straightforward to show that Goodman and Kruskal’s *τ*_*c*_ and *τ*_*r*_ [37] are both equivalent to *ϕ*^2^ for 2 × 2 tables. These equivalences confirm that Pearson’s *χ*^2^, *V*^2^, *τ*_*c*_ and *τ*_*r*_ are composite statistical quantities that average over alternative forms of variation and are therefore subject to ambiguous interpretation. The $R^{r(c-1)}\mapsto R^{1}$ mappings consist of multidimensional sums and products across rows and columns, resulting in confounding effects because of dependence between them.

In the absence of an engineering or functional model, the specification of a vector basis for proportional variation for an *r* × *c* table is not a well-posed problem [39]; i. e., there isn’t a unique solution. This constitutes a fundamental limitation for the formulation of an effect size measure. Consider a two-component proportional system represented by vectors, **u**, **v** ∈ △^*N*^ with *N* > 1, and $u,v\in R^{N+1}$. The two default center-of-mass vectors are ***μ*** = (**u** + **v**)/2, and ***δ*** = (**u** − **v**)/2. However, there isn’t a standard procedure for choosing the additional 2*N* − 2 vectors needed to form a complete basis. Alternatively, a single coordinate or a sum of coordinates could serve as the basis for estimating an effect size. This corresponds to choosing a △^1^ × △^1^ subspace for the representation of proportional variation; e. g., *δ* = (*u*_*i*_ + *u*_*j*_) − (*v*_*i*_ + *v*_*j*_), with {(*u*_*i*_ + *u*_*j*_, 1 − *u*_*i*_ − *u*_*j*_), (*v*_*i*_ + *v*_*j*_, 1 − *v*_*i*_ − *v*_*j*_) ∈ △^1^}. A representation of the 2*N* degrees of freedom for a two-component △^*N*^ × △^*N*^ system would require the specification of *N* 2 × 2 tables. Therefore, the 2 × 2 table serves an elementary role in the decomposition of multiproportional variation due to the minimal properties of △^1^. The recommended approach is to adopt a multidimensional representation of proportional variation and “reduce any multiple-level or multiple-variable relationship to a set of two-variable relationships” [25]. Similar advice has been given for avoiding the compounding effect of the ANOVA null hypothesis, to break down “complicated hypotheses into smaller, more easily understood pieces” [40]. Ways in which an *r* × *c* table might be partitioned and marginalized have been described by Kateri [41]. The objective is to construct a set of 2 × 2 tables that encompass relevant forms of proportional variation for the particular application. This multidimensional representation should be combined with the specification of cost-benefit trade-offs in assessing the effect size for proportional variation. In the next section, we discuss the use of 2 × 2 tables in the CART algorithm. However, high-dimensional search is still a developing area [42, 43], and a detailed assessment of the pros and cons for various approaches is beyond the scope of this paper.

### 1.7 Gini information gain and *ϕ*^2^

In this section, we examine connections between effect size and information gain (IG) measures used in standard implementations of the CART algorithm. CART creates a binary decision tree by the recursive partitioning of the association between response and independent variables [44–46]. Each node of the tree corresponds to a binary partition of the range of an independent variable. Each terminal node is a classification identified by a unique combination of intervals of the independent variables. In standard implementations, the partition parameters for a node are determined by maximizing IG for the response variable in an exhaustive search of associations over all independent variables. In each iteration, the set of statistics obtained for the binary partitions of an independent variable constitutes a CART association graph. Our objective is to compare CART graphs for effect sizes including IG. To simplify the discussion, we consider the case where the response variable is binary. Then, the data for a partition correspond to a 2 × 2 table [47]. Then, IG is defined as the parent node impurity, *I*(*S*), minus the weighted impurities for the subnodes *I*(*S*_1_) and *I*(*S*_2_),

$$\begin{array}{c} \text{IG}\left(S_{1},S_{2}\right)=I\left(S\right)-x_{1}I\left(S_{1}\right)-x_{2}I\left(S_{2}\right), \end{array}$$ (23)

where the weight factor is $x_{i}=\frac{n_{i}}{n}$, *n*_*i*_ is the number of elements in node *S*_*i*_, and *n* = *n*_1_ + *n*_2_. Two popular impurity measures are the entropy, *E* = −∑*p*_*j*_lnp_*j*_, and Gini impurity, $G=1-\sum p_{j}^{2}$, where *p*_*j*_ is the proportion of class ‘*j*’ items in a set [16]. For a binary proportion vector, $\left(p_{m/n},p_{n/m}\right)=\frac{1}{m+n}\left(m,n\right)$, and the Gini impurity becomes *G*(*p*_*m*/*n*_, *p*_*n*/*m*_) = 2*p*_*m*/*n*_ *p*_*n*/*m*_. However, the *x*_*i*_ are subject to the same limitations as the weight factors for *s*_*M*_ (Eqs 9 & 10), and both IG_*E*_ and IG_*G*_ depend on the marginal sums. More concretely, we show that IG_*G*_ and *ϕ*^2^ are equivalent in CART. Let the rows and columns of Table 1 correspond to the subnodes and categories for the response variable, respectively. Then, *G*(*S*) for the parent node is

$$G(S)=2\frac{\left(a+c)(b+d\right)}{{\left(a+b+c+d\right)}^{2}}.$$

*G*(*S*_1_) and *G*(*S*_2_) are calculated from proportions for the row vectors (*a*, *b*) and (*c*, *d*), respectively. Then,

$$\begin{array}{cc} {\text{IG}}_{G}\left(S_{1},S_{2}\right) & =\frac{2}{a+b+c+d}[\frac{\left(a+c)(b+d\right)}{a+b+c+d}-\frac{ab}{a+b}-\frac{cd}{c+d}],\\ & =G\left(S\right)\phi^{2}, \end{array}$$ (24)

with substitution of the *ϕ* coefficient from Eq (17). Since *G*(*S*) is a constant for binary partitions at a parent node, we conclude that IG_*G*_ is equivalent to *ϕ*^2^. This confirms that IG_*G*_ depends on marginal sums due to the *x*_*i*_, in which the normalization factor *N* = *a* + *b* + *c* + *d* does not distinguish between rows and columns. Information gain measures of the form Eq (23) will be subject to this limitation, including IG_*E*_. It is known that IG_*E*_ and IG_*G*_ yield very similar results in CART [48], which confirms that IG_*E*_ is subject to dependence on marginal sums (Table 4). The limitations of IG_*G*_ raise the question of whether the column sum invariant *δ*_*c*,*a*−*c*_ statistic might be more appropriate for CART, which we consider in the next section.

**Table 4:** Classification tree partitions for NHC ‘short-stay rehospitalized’ data.

Impurity Measure	Split Value	*S*_**1**_ N, (1 star, 5 star)	*S*_**2**_ N, (1 star, 5 star)
**IG**_***G***_, **IG**_***E***_	22.0	1966, (0.41, 0.59)	2077, (0.58, 0.42)
***δ***_***c*,*a*−*c***_, **lower**	13.3	301, (0.30, 0.70)	3742, (0.51, 0.49)
***δ***_***c*,*a*−*c***_, **upper**	32.6	3874, (0.49, 0.51)	169, (0.71, 0.29)

CART association split value, sample size (N), and (1-star, 5-star) proportions for subnodes *S*_1_ and *S*_2_, for Gini (IG_*G*_) and entropy (IG_*E*_) information gain, and proportion difference (*δ*_*c*,*a*−*c*_).

## 2 Data analysis and results

### 2.1 Data preparation

**Fig 6:**
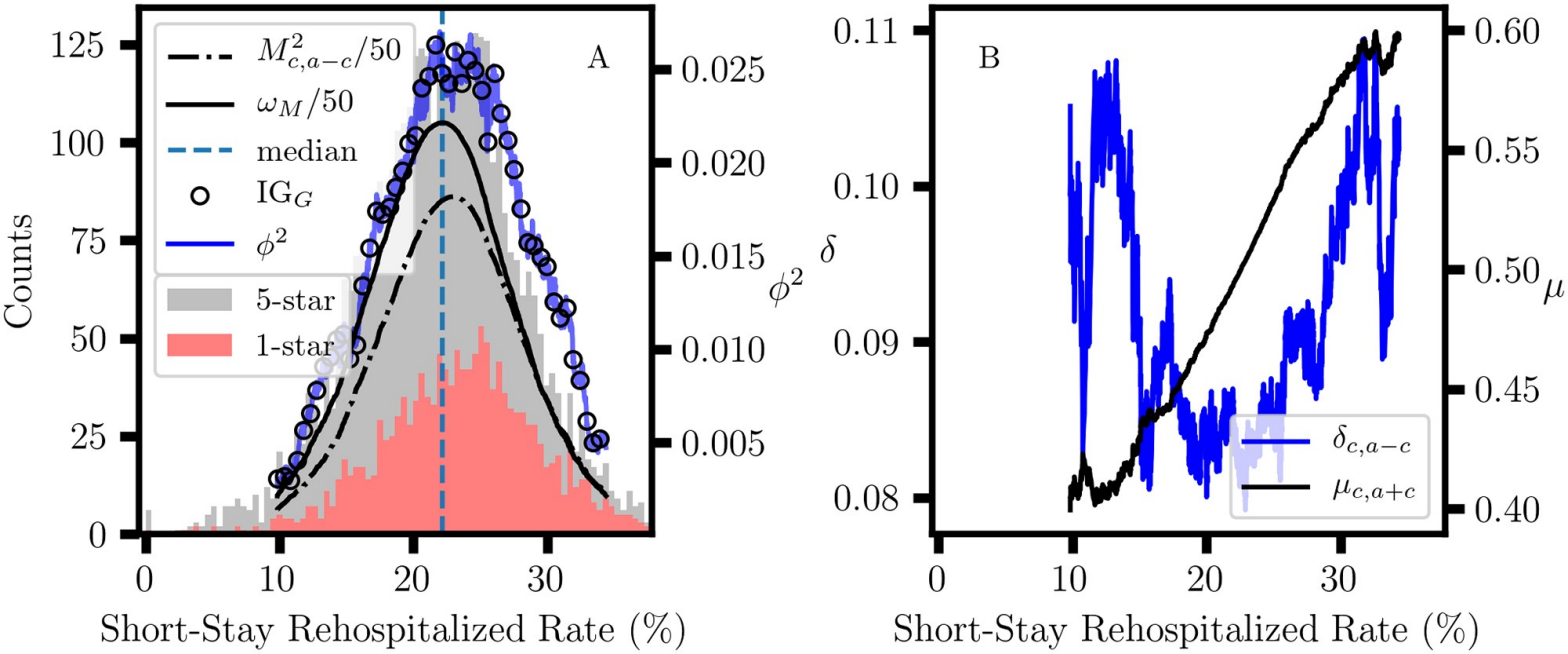
CART association graph. A: Stacked histograms for First Quarter, 2018 ‘Percentage of short-stay residents who were rehospitalized after a nursing home admission’ data for nursing homes with a 1 star or 5 star overall rating; the dashed line is the median value. CART associations between Nursing Home Compare ‘Rehospitalized’ quality measure and ‘1 star, 5 star’ overall rating for IG_*G*_ and *ϕ*^2^ are also shown. IG_*G*_ was scaled to match *ϕ*^2^. Both $M_{c,a-c}^{2}$ and *ω*_*M*_ were scaled by 1/50. B: Column scaling invariant center-of-mass coordinates, (*δ*_*c*,*a*−*c*_, *μ*_*c*,*a*+*c*_), for the two-component proportional variation in the standard one-simplex, △^1^.

The Centers for Medicare and Medicaid (CMS) conduct regular inspections of nursing homes to assess compliance with regulations and survey residents to assess the quality of patient care. The CMS quality measures data and Five-Star rating assignments are publicly available from the Nursing Home Compare (NHC) website [49]. The analysis of NHC data is an important problem in itself [50–52] and is the subject of our ongoing work [53]. Nursing homes are dynamic systems where the measurement of performance is essential for managing cost, but this constitutes a complex problem for which there is not a unique or ‘best’ solution. The challenge is to develop data analysis methods that can help identify public health criteria for classifying the quality of patient care in nursing homes, or some approximation thereof. However, in this work our interest is limited to the comparison of CART association graphs for effect size measures. First Quarter, 2018 NHC data for eighteen quality measures were retrieved, selecting only those nursing homes with either a 1 star or 5 star overall rating, corresponding to 1394 and 2649 nursing homes, respectively. Selecting ‘1 star, 5 star’ rating data creates a binary response data set, which is convenient for our purpose; otherwise, data for all five ratings would be included in the CART analysis. The distributions of NHC ‘Percentage of short-stay residents who were rehospitalized after a nursing home admission’ (Rehospitalized) quality measure data for 1 star and 5 star overall ratings are broad and largely overlap (Fig 6A). This result implies that the *M*_*i*_ for the corresponding contingency tables will tend to be much less than 1, as required for our demonstration.

### 2.2 Effect size in CART

In demonstrating the marginal sum dependence of various effect size measures, we must choose an elementary contingency table analysis problem. CART analysis for a binary response variable (bCART) is well suited for this purpose. In searching for an optimal binary partition of an independent variable, bCART generates a set of 2 × 2 tables where the sample sizes, *n*_1_ and *n*_2_, of the two subnodes vary over almost the entire range of the fixed sum *N* = *n*_1_ + *n*_2_; a minimum size is usually specified because a partition where either of the subnodes is too small is not informative. We let the rows and columns of Table 1 correspond to the two subnodes and the ‘1 star, 5 star’ rating for the response variable, respectively. Effect size results for a bCART scan for association between the Rehospitalized quality measure and NHC ‘1 star, 5 star’ overall rating are shown in Fig 6. The exact match between IG_*G*_ and *ϕ*^2^ (Fig 6A) is consistent with Eq (24) because *G*(*S*) is constant. The parabolic variation of *ϕ*^2^ is explained by Eq (21) because the variation in the marginal sum factor, *M*_*c*,*a*−*c*_, outweighs the much smaller variation in the proportional effect, *δ*_*c*,*a*−*c*_ (Fig 6B). The parabolic variation of $M_{c,a-c}^{2}$ is in turn explained by the approximate similarity with *ω*_*M*_. Replacing each marginal sum in Eq (18) by the corresponding proportion yields

$$\begin{array}{cc} \omega_{M} & =\frac{p_{a+b}p_{c+d}}{p_{a+c}p_{b+d}},\\ & =\frac{p_{a+b}(1-p_{a+b})}{p_{a+c}\left(1-p_{a+c}\right)}. \end{array}$$

**Fig 7:**
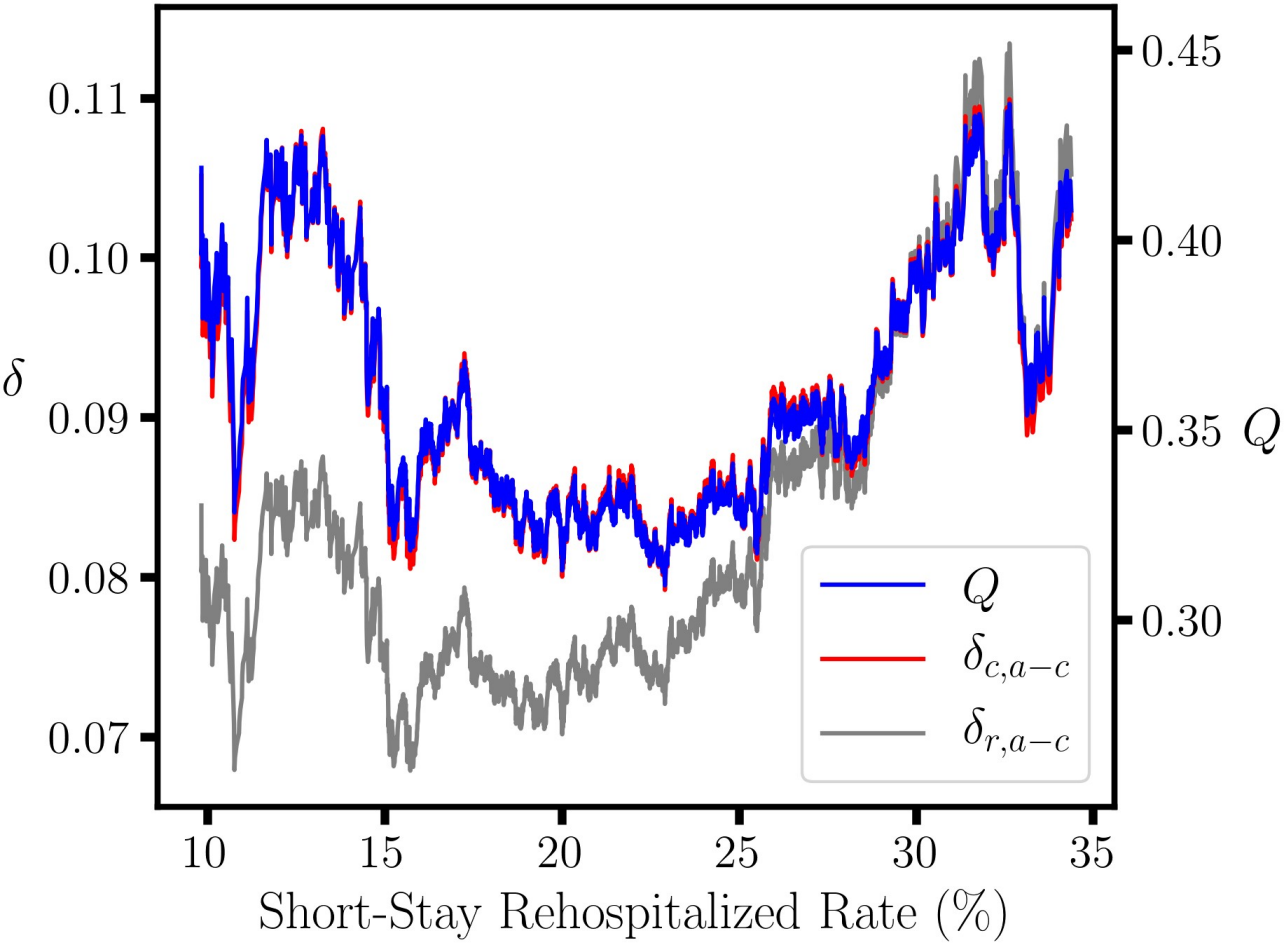
Scaling invariant effect size statistics for CART. Yule’s *Q* and *δ*_*c*,*c*−*a*_ yield similar results in the CART association between the First Quarter, 2018 Nursing Home Compare ‘Rehospitalized’ data and ‘1 star, 5 star’ overall rating. *δ*_*c*,*c*−*a*_ and *δ*_*r*,*c*−*a*_ are the column and row scaling invariant proportion differences, respectively. *Q* is invariant to scaling of either columns or rows.

The denominator corresponds to the binomial variance for the parent set, which is constant. The numerator corresponds to the binomial variance for subnode size proportions, (*a* + *b*): (*c* + *d*), so *ω*_*M*_ has a maximum when *a* + *b* = *c* + *d*, which coincides with the median Rehospitalized value. Consequently, the parabolic dependence of *ϕ*^2^, with the maximum near the median value, largely reflects the variation in the subnode sample size instead of ‘1 star, 5 star’ composition. In contrast, *δ*_*c*,*a*−*c*_ is column sum invariant and yields very similar results to Yule’s *Q*, which is invariant to scaling of either rows or columns (Fig 7); the correlation is higher than 0.99 for 16 NHC quality measures, and the lowest is 0.91. Note that this similarity does not represent a special relation with *Q* and results from the numerical properties of *δ*_*c*,*a*−*c*_ and *μ*_*c*,*a*+*c*_ for these data (Eq 4). The lower correlation (*r* = 0.78) between *δ*_*c*,*a*−*c*_ and *δ*_*r*,*a*−*c*_ confirms that different forms of proportional variation can be distinguished; *δ*_*r*,*a*−*c*_ also measures the difference in subnode composition but is row sum invariant. The U-shaped *δ*_*c*,*a*−*c*_ association graph has two maxima, so there are two possible CART partitions (Table 4). The relatively small subnode with Rehospitalized below 13.3% is enriched in the 5 star rating, corresponding to better than average patient care. Above 32.6%, the patient care is worse than average because it is associated with enrichment of the 1 star rating. The middle range from 13.3-32.6% includes the majority of nursing homes with average performance. In comparison, IG_*G*_ and IG_*E*_ produce subnodes that are nearly equal in size and with much lower degrees of enrichment in the ‘1 star, 5 star’ proportions. Thus, *δ*_*c*,*a*−*c*_ is more effective than IG_*G*_ and IG_*E*_ in identifying partitions that correspond to a difference in the ‘1 star, 5 star’ composition.

**Fig 8:**
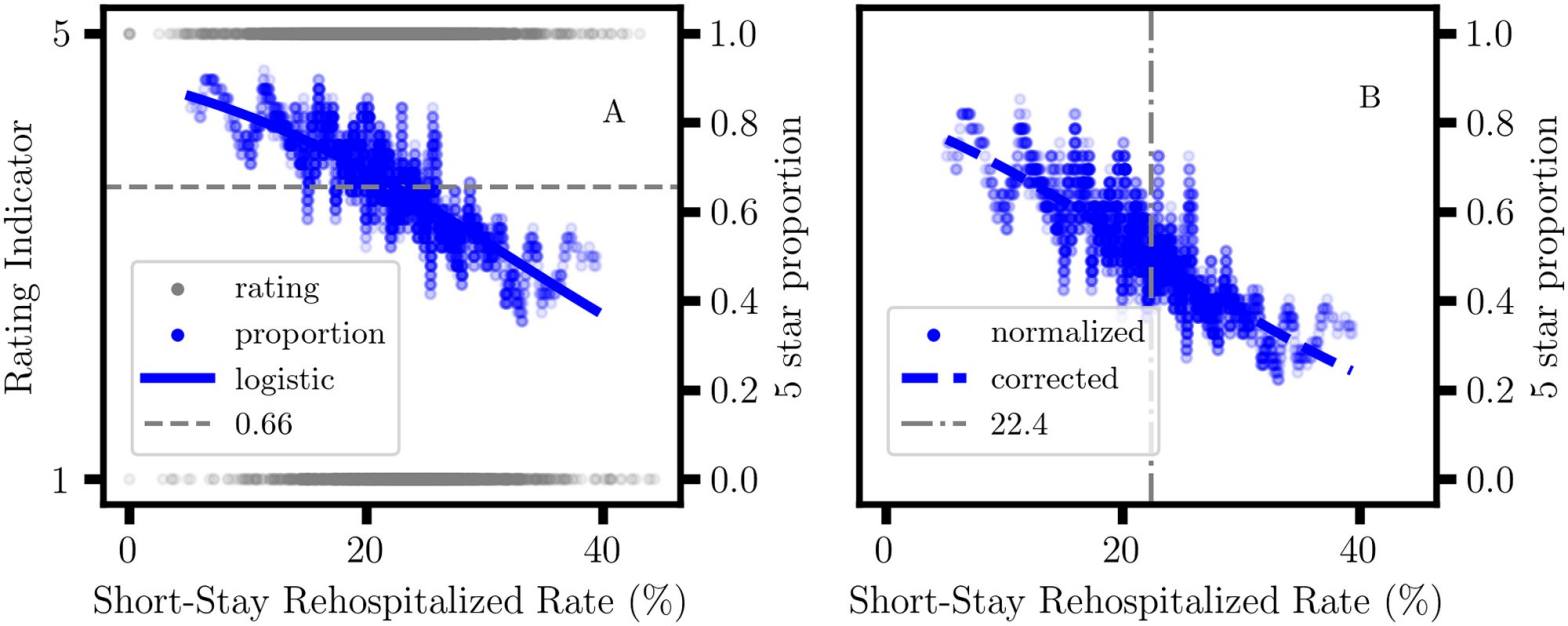
Sample size effects in logistic regression. A: Logistic model for Nursing Home Compare ‘Rehospitalized’ data and ‘1 star, 5 star’ overall rating. The moving average ‘5 star’ proportion is included for reference. The ‘5 star’ sample size proportion, $\frac{n_{5}}{n_{1}+n_{5}}=0.66$, is shown as a horizontal line; *n*_1_ = 1394, and *n*_5_ = 2649. B: Normalized ‘5 star’ proportion adjusted for unequal sample sizes, and the adjusted logistic curve. The midpoint value, *x*_0_ = 22.4, for the logistic curve is shown as a vertical line; the ‘Rehospitalized’ median is 22.2.

The logistic regression method provides a graphical view of the effect of sample size parameters on proportional variation in categorical data analysis (Fig 8A). The ‘1 star, 5 star’ rating data were analyzed using the LogisticRegression function in the scikit-learn library with the ‘lbfgs’ solver [54]. A moving average of the ‘5 star’ rating proportion is included in the graph as a reference for the logistic curve. The normalized ‘5 star’ proportion adjusted for inequality in the ‘1 star, 5 star’ sample sizes and the corresponding adjusted logistic curve are shown in (Fig 8B). The variation in proportion confirms that the left and right tails of the Rehospitalized distribution correspond to nursing homes with above and below average performance, respectively, consistent with the CART association results. The logistic model for the ‘5 star’ proportion, $y=\frac{c_{5}}{c_{1}+c_{5}}$, is usually expressed as

$$\begin{array}{c} y=\frac{1}{1+e^{-(a+bx)}}, \end{array}$$ (25)

where parameters, (*a*, *b*), are determined from the curve fit. The adjustment for the logistic curve was obtained using the change in coordinates

$$\begin{array}{c} a=-bx_{0}-\text{ln}(\frac{n_{1}}{n_{5}}), \end{array}$$

where *n*_1_ and *n*_5_ are the sample sizes for the 1 star and 5 star ratings in the data set, respectively. Substitution into Eq (25) yields

$$\begin{array}{c} y=\frac{1}{1+\frac{n_{1}}{n_{5}}e^{-b(x-x_{0})}} \end{array}$$

such that $y\left(x_{0}\right)=\frac{n_{5}}{n_{1}+n_{5}}$. In a data set where *n*_1_ = *n*_5_, *y*(*x*_0_) = 1/2, and *x*_0_ correspond to the mid-point value for the logistic curve. Then, there are two sample-size-independent parameters, *b* and *x*_0_.

## 3 Discussion

The renewed warnings from the statistics community about the limitations of statistical significance methodology has created a perplexing situation, given that there is a wide range of opinion on the underlying causes and solutions [55, 56]. Claims have also been made about effect size [25, 26, 57] as a better alternative, but the lack of consensus on the utility of commonly used association coefficients, such as the odds ratio [8, 10], the simple matching coefficient and *ϕ* [5, 11], hinders development of this approach. In this paper, we describe a rigorous framework for representing proportional variation in a 2 × 2 table, which helps in resolving the marginal sum dependence problem for association coefficients. We show that a 2 × 2 table is associated with four forms of proportional variation resulting from the factorization as a product of proportion and diagonal row or column sum matrices. Association coefficients, such as *ϕ*, the odds ratio, and the simple matching coefficient, which do not distinguish between rows or columns, correspond to averages of proportional effects and lack clear interpretation. The two-component structure implies that there are two degrees of freedom corresponding to the displacement of two point vectors in the standard one-simplex, △^1^. An effect size measure then requires the specification of a perspective function of the center-of-mass coordinates, (*δ*, *μ*), which is potentially unique for each application because of differences in cost-benefit trade-offs. In practice, classification problems vary widely in difficulty depending on the degree of overlap between the underlying distributions. Fisher’s irises data set [58] is an example of a classification problem for well separated distributions, where different association coefficients achieve similar results because of degeneracy, particularly when the 2 × 2 table is diagonally symmetric or the effects are highly correlated. Conversely, differences in performance between association coefficients are best observed when the underlying distributions overlap. We also show that both Gini and entropy information gain are subject to dependence on marginal sums, which degrades the performance of the CART algorithm. Alternatively, the proportion difference with marginal sum invariance for the response variable provides a significant improvement in the performance of the CART algorithm. We conclude that the results in this paper demonstrate that equalization of either row or column sums of a 2 × 2 table serves as a correction for unbalanced sample sizes, as suggested by Goodman and Kruskal [2].
